# Solid-state synthesis of CdFe_2_O_4_ binary catalyst for potential application in renewable hydrogen fuel generation

**DOI:** 10.1038/s41598-022-04999-1

**Published:** 2022-01-31

**Authors:** Abdullah M. Asiri, Waheed A. Adeosun, Sher Bahadar Khan, Khalid A. Alamry, Hadi M. Marwani, Shaik M. Zakeeruddin, Michael Grätzel

**Affiliations:** 1 Center of Excellence for Advanced Materials Research, King Abdulaziz University, P.O. Box 80203, Jeddah, 21589 Saudi Arabia; 2 Department of Chemistry, King Abdulaziz University, P.O. Box 80203, Jeddah, 21589 Saudi Arabia; 3 Laboratory of Photonics and Interfaces, École Polytechnique Fédérale de Lausanne, 1015 Lausanne, Switzerland

**Keywords:** Chemistry, Engineering

## Abstract

Clean energy is highly needed at this time when the energy requirements are rapidly increasing. The observed increasing energy requirement are largely due to continued industrialization and global population explosion. The current means of energy source is not sustainable because of several reasons, most importantly, environmental pollution and human health deterioration due to burning of fossil fuels. Therefore, this study develops a new catalyst for hydrogen and oxygen evolution by water splitting as a potential energy vector. The binary metal oxide catalyst CdFe_2_O_4_ was synthesized by the solventless solid-mechanical alloying method. The as-prepared catalyst was well characterized by several methods including field emission scanning electron microscopy (FESEM), X-ray diffraction spectroscopy (XRD), X-ray photoelectron spectroscopy (XPS), Fourier Transform infrared red spectroscopy (FTIR), energy dispersive X-ray spectroscopy (XEDS). The as-prepared catalyst, CdFe_2_O_4_ was successfully applied for water electrolysis at a moderate overpotential (470 mV). Specifically, the onset potential for the oxygen and hydrogen evolution reactions (OER and HER) were 1.6 V_/RHE_ and 0.2 V_/RHE_ respectively (vs. the reversible hydrogen electrode). The electrode potential required to reach 10 mA/cm^-2^ for OER (in alkaline medium) and HER (in acidic medium) was 1.70 V_/RHE_ (corresponding to overpotential η = 0.47 and − 0.30 V_/RHE_ (η = − 0.30 V) respectively. Similarly, the developed OER and HER catalyst displayed high current and potential stability for a period of 12 h. This approach is seen as the right track of making water electrolysis for hydrogen energy feasible through provision of low-energy requirement for the electrolytic process. Therefore, CdFe_2_O_4_ is a potential water splitting catalyst for hydrogen evolution which is a clean fuel and an antidote for world dependence on fossil fuel for energy generation.

## Introduction

Clean energy sources attract much needed attention these days as the effect of environmental pollution becomes more evident. The combustion of fossil fuels has been the major source of energy supply and has been implicated with environmental pollution resulting in the release of serious contaminants such as nitrous and sulfur oxides (NO_x_, SO_x_). The environmental problems combined with dwindling fossil fuel supply has called for a sustainable and clean energy source. Electrochemical water splitting using electricity generated by solar energy conversion or other environment-friendly processes is a process capable of generating a huge amount of hydrogen as a fuel cell carrier gas and industrial processes essential feedstock^1^. Although there are several ways of hydrogen generation, for example by natural means and wet synthesis (chemical method)^2^, electrochemical hydrogen generation provides carbon-free hydrogen at a large volume suitable for industrial continuous use. Hydrogen gas generation from water electrochemical splitting suffers huge setbacks in practical application because of the high overpotential needed for the process^3^. The use of noble metals as electrocatalysts is associated with a low overpotential, but its high cost and limited availability for industrial use make them unattractive. Several efforts have been channeled towards the development of inexpensive and effective catalysts to perform the hydrogen and oxygen evolution reactions.

For instance, Liu et al. reported use of bimetallic 2D-MOF for oxygen evolution reaction. In their study, stoichiometric ratios of Ni and Co were harnessed for synthesis of bimetal 2-methylimidazole based 2D-MOF. The overpotential to achieve 10 mA/cm^2^ in alkaline solution was 310 mV. The catalyst also showed high stability^4^. In another study by Djara et al., lanthanum series based composite was exploited for overall water splitting application. The electrocatalysts made up of polyaniline-iridium and polyaniline-ruthenium had overpotential of 36 mV (HER) and 240 mV (OER) for generating 10 mA/cm^2^ respectively. The observed low overpotential was attributed to the synergistic effect of conducting polyaniline and iridium and ruthenium^5^. Phosphides have also been explored for application in electrochemical water splitting. For instance, Kumar et al. developed nickel phosphide on carbon support. In other study, low overpotential of HER and OER reactions were recorded (137 and 360 mV) respectively at 10 mA/cm^2^ current density^6^. In addition, non-noble metal based electrocatalysts using solvothermally synthesized Ni_3_S_2_ was reported for water splitting application. Although the catalyst had a relatively high overpotential of 660 mV and 350 mV for OER and HER respectively, the catalyst displayed very high stability^7^. In another study by Yang et al., Fe_2_O_3_ was doped with Co_3_O_4_ by hydrothermal method. The synergistic effect between the transition metals and unpaired d-orbitals in Fe and Co ensures efficient charge transfer on the composite. This resulted in improved catalytic performance towards oxygen evolution reaction. In another effort by Xie et al. Co_3_O_4_-MnO_2_-CNT nanocomposite was synthesized and applied for water splitting application. The result of their studies indicate that oxygen evolution occurred with a current density of 10 mA/cm^2^ at overpotential of 500 mV with Tafel slope of 58 mV/dec^8^. Likewise, Wang et al. reported synthesis of CoFe_2_O_4_ based catalyst by wet chemical approach for use as OER catalyst. They claimed that an overpotential of 540 mV was required to attain current density of 10 mA/cm^2^. The onset potential for the OER was reported to be 1.64 V.

While great efforts have been expended on development of cheap, effective non-noble metal based water splitting catalysts, most of the developed candidates suffer setback in terms of high overpotential, cumbersomeness synthesis, environmental unfriendly method of synthesis and finally, long term instability of their performance. The trend of OER and HER electrocatalysts development warrants highly effective, environment friendly, cheap and stable electrocatalysts and this phenomenon informed the current study. Therefore, in this work, a binary non-noble metal-based catalyst (CdFe_2_O_4_) was developed to provide cheap, effective and stable water splitting catalysts. In this new approach increased active sites exposure of Cd/Fe_2_O_4_ as well as synergistic effect between the two transition metals is believed to have aided the charge transfer process, involved in the OER reaction to proceed at low over-potentials. The insight into this binary material (Fe_2_O_3_/CdO) was conceived as a result of excellent catalytic and conductivity of the constituent composite. For instance, CdO is n-doped material due to the presence of oxygen vacancies^9^. Likewise, Fe_2_O_3_ being a weakly ferro-magnetic material based on its unpaired d-electron in Fe^3+^, supports variable oxidation states of the precursor metal ion, thereby increasing its redox-catalytic property. Equally important to the nature of electrocatalysts for water splitting is the method of catalyst synthesis. Several materials such as chalcogenides^10^, nitrides^11^, sulfides^12^, carbides^13^, phosphides^14^, and oxides^15^ have been explored for electrochemical water splitting process. Oxides are the most promising because of their catalytic function and ease of synthesis. Conventionally, the major means of metal oxides (MOx) synthesis are solvo-thermal method^16^, electrochemical method^17^, chemical precipitation method^18^, hydrothermal method^19,20^, sputtering method, laser-deposition, chemical vapour deposition etc. However, lately solid-state synthesis of MOx is gaining much attention because of its ability to generate large surface area of nanoparticle, and less environmental footprint (solventless reaction). In addition, pryrolitic treatment of the synthesized composite ensures elimination of likely interferents/inbitors and promotes orderliness of the composite interfaces. Other studies have indicated that ordered heterostucture ensures provision of more active sites, which improves overall water splitting efficiency^21^. Therefore, this study aims to design a composite with ordered heterostucture having activated large surface area for electrochemical water splitting at low overpotential comparable to noble-metal based catalysts.

To the best of our knowledge, this study describes solventless synthesis of the CdFe_2_O_8_ mixed oxide heterostucture for the first time. This study also applies this novel material as catalyst for OER and HER reactions at moderate overpotentials (0.47 V and 0.3 V to drive 10 mA/cm^2^) for OER and HER respectively. The synthesized CdFe_2_O_4_ also displayed good stability over an electrolysis period of 12 h.

## Experimental

### Reagents & apparatus

The reagents used for this experiment include: Iron (II) chloride (FeCl_2_) (Sigma-Aldrich, USA); cadmium (II) nitrate (Cd(NO_3_)_2_) (Sigma-Aldrich, USA); sodium hydroxide (Sigma-Aldrich, USA); sodium dihydrogen phosphate (Sigma-Aldrich, USA), disodium hydrogen phosphate (Sigma-Aldrich, USA), potassium hydroxide (Sigma Aldrich, USA), sulfuric acid (Sigma-Aldrich, USA); nafion solution (dissolved in 5% ethanolic) and distilled water. All the reagents used are analytical grade and were used as purchased. Moreover, the apparatus used for this study include mortal and pestle, muffle furnace (model), powder X-ray diffraction (XRD) spectrometer (model), field emission scanning electron microscope (FESEM), Fourier transform infrared spectrometer (FTIR). In addition, electrochemical measurements (water splitting studies) were conducted with an electrochemical workstation (Autolab Potentiostat AUT83887) connected to a three-electrode cell—working, reference and counter electrode. The working electrode at any point in time is bare gold electrode (BGE) (1.6 mm diameter) or CdFe_2_O_4_ deposited on a gold electrode. Reference electrode is made up of Ag/AgCl (in 3 M KCl), henceforth to be reported as AgCl reference electrode, while the counter electrode is a platinum wire (1 mm diameter).

### Preparation of CdFe_2_O_4_ ternary composite

Solid-state synthesis approach was applied by taking a stoichiometric quantity of Cd(NO_3_)_2_, FeCl_2_ and NaOH in ratio 1:1:1. The measured chemical components was grinded and well homogenized using lab-grade mortar and pestle. The grinding was maintained for a period of 30 min to allow proper intercalation of Cd^2+^ and Fe^2+^ precursors. After a visibly change in coloration from light brown to black, fine textured and well-mixed mixtures were obtained. The precursor mixture was then subjected to pyrolytic treatment in a muffle furnace at 600 °C for 4 h. After pyrolytic treatment, the precursor mixtures turned dark-grey and was kept at room temperature until use.

### Characterization procedures

The synthesized CdFe_2_O_4_ binary composite was characterized using several techniques. The crystallinity and structure orderliness of the synthesized materials was investigated with powder X-ray diffraction (XRD) (Arl Xtra) using Cu-Kα (radiation source) at a scan rate of 5° min^−1^ and 2θ range of 15° to 80°. The morphological study was conducted using SEM (JEOL, JSM-7600F) which was fitted with EDX (Oxford) for elemental composition investigation. The binding energy and elemental composition was also assessed by X-ray photoelectron spectroscopy (XPS) (Kα 1, 1066). The functional group (metal-oxide, M–O) was investigated by Fourier transform infrared spectroscopy (FTIR) (Thermo-Scientific). Electrochemical characterization of the as-prepared CdFe_2_O_4_ was conducted by electrochemical impedance spectroscopy (EIS) and cyclic voltammetry (CV) using potentiostat (PGSTAT302N-AUT85887). For the EIS, the spectra frequency range was from 100 kHz to 1 mHz. The equivalent circuit diagram for the acquired EIS data was fitted by in-built Autolab EIS fit. The electron mobility in the as-prepared CdFe_2_O_4_ was also investigated with CV at a potential range of 0 V to 1.0 V using a scan rate of 100 mV and a step potential of 8 mV.

### Water splitting studies

The conditions of electrochemical experiments have been reported in Sect. 2.1. As regards the electrocatalyst preparation, 100 µg of the as-prepared CdFe_2_O_4_ was dispersed in ethanolic solution (10%). The dispersed materials was then casted on GE surface with the aid of 1 drop of liquid nafion. Linear sweep voltammetry (LSV) was employed for current–voltage measurements. For OER study, the supporting electrolyte used was 1 M KOH aqueous solution (purged before experiment). The LSV was conducted in the potential range of 0.6 V to 1.8 V (vs. Ag/AgCl), scan rate of 100 mV/s, amplitude of 5 mV and step potential of 8 mV. For HER study, the supporting electrolyte used was 0.5 M sulfuric acid aqueous solution. The LSV was conducted in the potential window of 0 V to − 1.5 V (vs. Ag/AgCl), scan rate of 100 mV. AC amplitude of 5 mV and step potential of 8 mV. The working electrode potential was converted to the relative hydrogen electrode (RHE) scale using the Nernst equation;

$${\text{E}}_{{/{\text{RHE}}}} \left( {\text{V}} \right)\, = \,{\text{E}}_{{/{\text{AgCl}})}} \, + \,0.{2}0{7} {\text{ V}}\, + \,0.0{59} {\text{ V}}\cdot{\text{ pH}}$$

where E_/RHE_ and E_/AgCl_ is the electrode potential vs. the reversible relative hydrogen electrode (RHE) and E_Ag/AgCl_ denotes working cell potential respectively.

### Faradaic efficiency for electrochemical water oxidation to oxygen

The amount of oxygen produced was quantified by gas chromatography (Trace GC Ultra, Thermo Scientific). The gas chromatography is equipped with a shincarbon micropacked column (Restek) for separation and a pulse discharge detector (Vici) for analyzing the amount of product. A 100 μL sample loop (Vici) was used and the gaseous aliquot was taken automatically via a 6-port switching valve (Vici). The oven was set at an initial temperature of 40 °C for 2 min, followed by a temperature ramp at 40 °C/min to 200 °C. The oven was then held at 200 °C for 2 min. Each run was 8 min and about 6 min is needed to cool down the oven for the next run. The gas chromatography was calibrated by injecting known concentration of oxygen gas dissolved in Helium matrix (99.9999%). The peak area was plotted against the concentration (in ppm) of oxygen, resulting in a linear fitting curve, (Figure S2).

The electrochemical oxygen evolution reaction was carried out in a custom-built two-compartment cell, made of Teflon material. The cathodic chamber was gas-tight and Helium gas (99.9999%) was used as the inert gas to purge out the evolved oxygen gas to the sample loop at the constant rate of 10 sccm during the electrolysis. The oxygen evolution was tested at 10 mA cm^−2^ for 120 min. 9 injections were taken during the electrolysis and the average of them (except the first one since it takes ~ 15 min to equilibrate the headspace) were used for calculating the average faradaic efficiency.

## Results and discussion

### Material characterization

**Figure 1:**
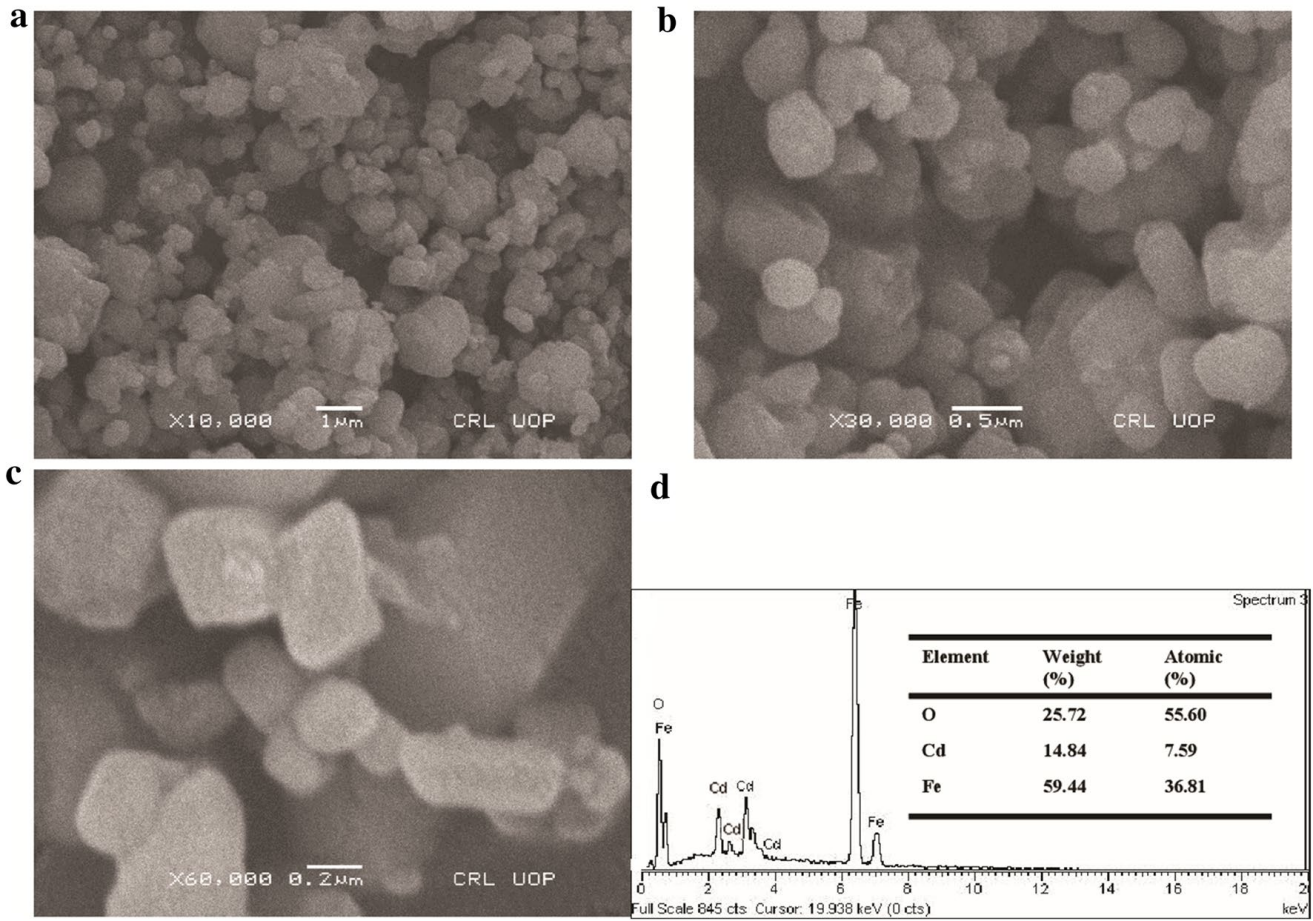
(**a**) FESEM image of CdFe_2_O_4_ (low magnification). (**b**,**c**) high magnification FESEM image. (**d**) XEDS spectrum of CdFe_2_O_4_.

The morphology of the as-prepared CdFe_2_O_4_ was investigated with FESEM and the obtained images are presented in Fig. 1a–c. As shown in the Fig. 1a, the low magnification reveals a mixture of nanostructures with varying shape majorly cuboid and rhombic phases. The higher magnification (Fig. 1b,c) shows well defined cuboid nanostructures. The edges of these nanostructures are suggested as potential active sites for the catalytic activity. The active sites in CdFe_2_O_4_ in addition to the O-vacancies, ensure excellent electron transport between the catalyst and the electrolyte. The cuboid and rhombic phases create a kind of defect on the catalyst surface which is ultimately needed for electron transport. The obtained FESEM image indicate the crystallinity nature of the synthesized materials and hence, suggesting that the choice method of synthesis yielded well defined nanocrystals.

In addition, the synthesized CdFe_2_O_4_ was investigated for elemental composition by XEDS. The obtained results are presented in Fig. 1d. The XEDS spectrum (Fig. 1d) reveals that the synthesized material was made up of cadmium, iron and oxygen. The obtained result is consistent with the elemental composition of CdFe_2_O_4_. Therefore, the obtained XEDS result confirms the material to be composed of cadmium oxide and iron oxide. Absence of any other peaks and/or element indicates absence of any impurity or contaminant in the material.

**Figure 2:**
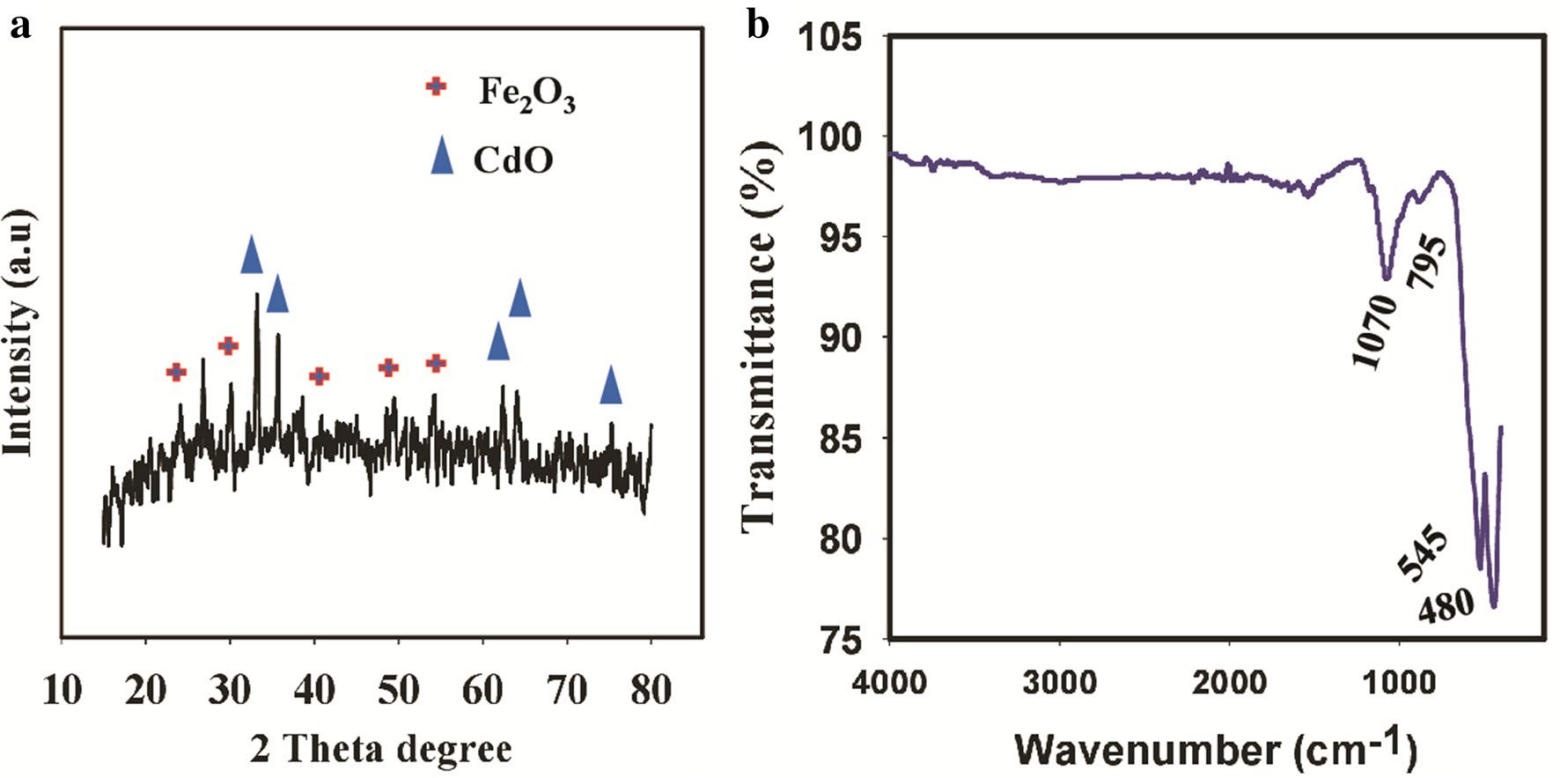
(**a**) XRD spectrum of CdFe_2_O_4_. (**b**) The obtained FTIR spectrum of CdFe_2_O_4_.

The structural investigation of the synthesized CdFe_2_O_4_ was conducted by XRD analysis and the obtained XRD spectrum is presented in Fig. 2a. The obtained spectrum indicate the prepared nanoparticle composite to be moderately crystalline. Due to the moderate calcination temperature of 550 °C, the crystallinity is relatively low but this could be improved with higher calcination temperature (700 °C and above). The XRD spectrum of CdFe_2_O_4_ presented in Fig. 2a showed characteristic peaks at 2θ angle of 20.2°, 30.1°, 35.11°, 37.2°, 43.09°, 52.14° and 57.39° corresponding to crystal phases of (111), (220), (311), (222), (400, (422) and (511) respectively which indicate a rhombic structure of Fe_2_O_3_ (PDF 89-2810; PDF No: 01-079-007; JCPDS Card No. 01-084-0307)^22^. Also, the observed peaks at diffraction angle 2 θ of 32.2°, 38.1°, 55.38°, 65.2° and 70.3° corresponding to the (111), (200), (220), (311) and (222) facets, respectively could be attributed to CdO cubic phases (JCPDS Card No. 05-0640^23^. The CdFe_2_O_4_ crystallite size was determined using Debye Sherrer equation^24^ as given below;

$$D = \frac{K\lambda }{{\beta \cos \theta }}$$

where the crystal size is denoted with D; K denotes a constant (0.9) while the X-ray wavenlength (0.154 nm) is represented by λ. The width measured at half maximum is dented by β while the Bragg's diffraction angle is denoted by θ. The obtained particle size was 60 nm as calculated from the above. equation.

The FTIR study was conducted to reveal the metal oxide functionalities in the synthesized material. The obtained FTIR spectrum is presented in Fig. 2b. The spectrum shows peaks at the fingerprint region of IR spectrum chart for metal oxides. Specifically, the peaks at 1050 cm^−1^ and 795 cm^−1^ may be assigned to metal oxygen stretch of Cd–O and Fe–O^23,25^. Likewise, the observed peak at 545 cm^-1^ could be ascribed to stretching vibrations of CdO while the obtained peak at 480 could be attributed to Fe–O (Fe_2_O_3_)^22,26^.

### CdFe_2_O_4_ catalyst performance for OER reaction

**Figure 3:**
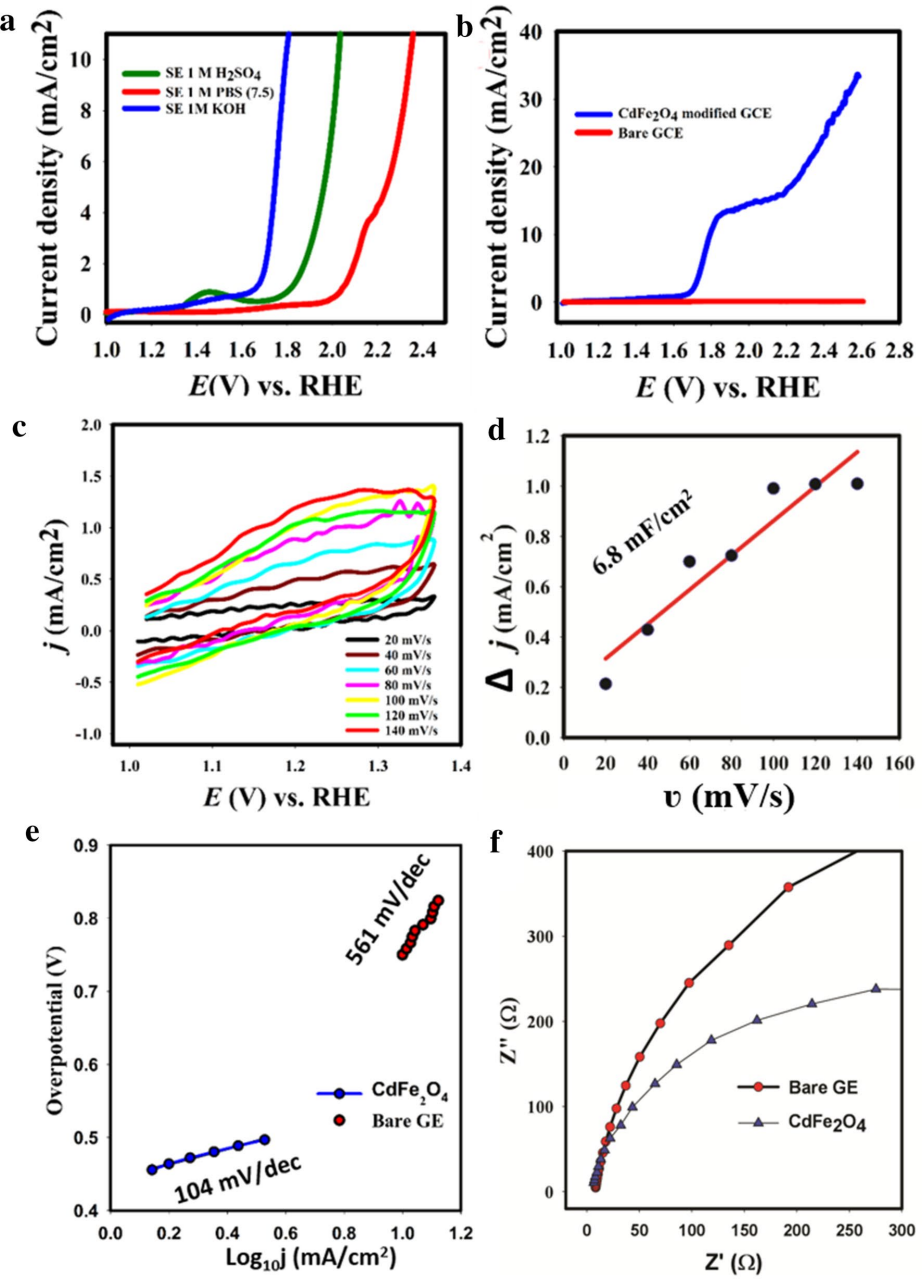
OER reaction studies: (**a**) Supporting electrolyte optimization. (**b**) Control study. (**c**) Effect of scan rate on OER. (**d**) Double layer capacitance (Cdl) determination (**e**) Tafel slope for OER reaction. (**f**) EIS spectrum of CdFe_2_O_4._

Supporting electrolyte optimization was first conducted by using different electrolyte of different pH medium such as 1 M KOH, 1 M PBS (pH 7.0) and 0.5 M sulfuric acid. The obtained linear sweep voltammogram for OER activity optimization with different supporting electrolytes is presented in Fig. 3.

The smallest onset potential (1.6 V vs. RHE) (overpotential 0.371 V if the electrolyte is saturated with pure oxygen at 1 bar) for OER reaction was achieved with 1 M KOH. The least effective SE was PBS solution, having highest onset potential (2.0 V vs. RHE). (Overpotential 0.77 V if the electrolyte is saturated with pure oxygen at 1 bar). In addition, the least overpotential to drive 10 mA/cm^2^ (0.47 V) was achieved with 1 M KOH, indicating the optimum condition for the OER. This current density corresponded to a turnover frequency (TOF) of 0.01 s^−1^.

Therefore, 1 M KOH was selected as the optimum SE. In an alkaline medium, oxidation of oxygen is enhanced as a result of excess hydroxyl ion. However, in acidic medium, shortage of hydroxyl ion and presence of H^+^ makes water oxidation difficult and subsequently limit amount of oxygen evolved. This indicates that oxygen is liberated from the alkaline media at no overpotential. These phenomena buttresses the obtained optimization results.

The faradaic efficiency of oxygen produced on CdFe_2_O_4_ was evaluated using gas chromatography over two hours electrolysis at 10 mA cm^−2^. The calibration curve of oxygen for gas chromatography is given in Fig. S2 (ESI). The gas stream was sampled every 840 s and the faradaic efficiency of oxygen ranges from 96.5–102.6%. The average value was 99.86% with a standard deviation of 2.16%. (Table S2). This demonstrates that practically all the currents density was used for producing oxygen.

The obtained result is very comparable to performance of some commercially used OER noble-metal based catalysts such IrO_2._ In the previous study, the onset potential for present commercially used noble-metal OER catalyst, IrO_2_ had onset potential of 1.55 V and an overpotential of 0.49 V to drive a current density of 10 mA/cm^2^ in an alkaline medium^27^. The high efficiency of the as-prepared catalyst for OER could be attributed to large and exposed surface area of CdFe_2_O_4_, which was achievable due to the solid-state method of preparation. In Table S2a (ESI), list of highly effective OER catalysts reported in recent time are listed for comparison with CdFe_2_O_4_. CdFe_2_O_4_ compares very well with the mentioned catalysts, in terms of onset potential, and low overpotential (at 10 mA/cm^2^) as well as general high current density. Figure 3b presents the control study (in 1 M KOH) where the current density of CdFe_2_O_4_ was compared with bare GE. It could be observed that CdFe_2_O_4_ modified GE attained 10 mA/cm^2^ current density at 1.7 V while that of bare GE had a current density of 0.2 mA/cm^2^ at the same potential.

The electrochemical active surface area (ECSA) of the CdFe_2_O_4_ modified GE was also evaluated from the plot of scan rate against oxidation current (Fig. 3c,d). The plot of Δj against the scan rate was used to calculate the double layer capacitance (C_dl_) which is also correlated to the ECSA. Corresponding to Cdl = 6.8 mF/cm^2^, and assuming that for a flat electrode it is C_dl _= 30 μA/cm^2^, the electrode roughness factor (real electrochemical active area/geometrical area) is 227, corresponding to ESCA = 4.56 cm^2^ for the CdFe_2_O_4_ layer (with an electrode cross section of 0.0201 cm^2^).

Compared to the geometric area of the GE (0.07 cm^2^), CdFe_2_O_4_ catalyst increased the active surface area of the electrode by > 2 times fold. This higher ECSA makes exchange of anions /charges between the substrate and the electrolyte very easy which eventually leads to improved OER reaction, compared to bare GE^28–31^. Apart from the improved active sites of CdFe_2_O_4_ surface, Fe is a transition metals with partially filled d-orbitals. D-orbitals of Fe^2+^ and Fe^3+^ overlap and this promotes catalytic activity of Fe active sites.

**Figure 4:**
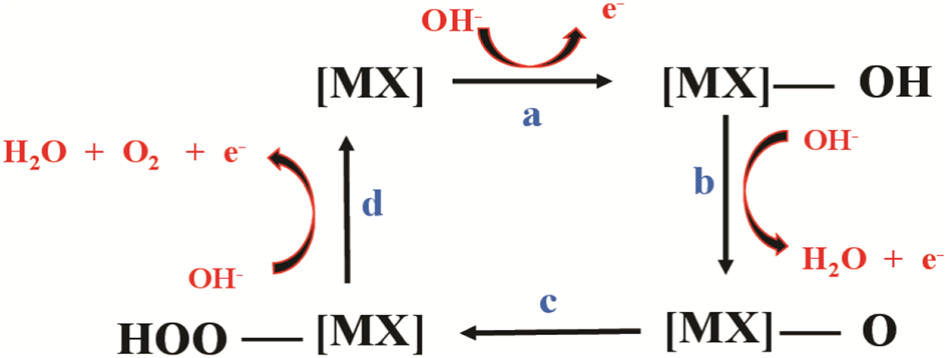
Suggested OER mechanism on metal oxides (MOx) catalyst. Where MX = CdFe_2_O_4_, a-d are reaction steps.

In addition, as shown in Fig. 3e, the Tafel slope for OER with CdFe_2_O_4_ was 104 mV/dec which indicates a faster reaction kinetics than bare GE with higher Tafel slope (458 mV/dec). The obtained Tafel slope OER CdFe_2_O_4_ catalyzed reaction compares well with the literature (Table S3a). The typical OER mechanism on metal oxides substrate (catalyst) is given in Fig. 4.

The above mechanism depicts the OER as multi-electron process as indicated in steps a-d. The obtained Tafel slope (104 mV/dec) suggests a three- electron transfer reaction for the OER.

Likewise, electrochemical characterization results in Fig. 3f also established improved electrochemical performance of CdFe_2_O_4_. Typically, in EIS, the charge transfer resistance (Rct) is represented by the semi-circle of the Nyquist plot; and the smaller the Rct, the better the electron/ion transfer on the substrate and electrolyte interface^32^. The Rct for CdFe_2_O_4_ was 343 Ω while that of bare GE was 596 Ω (Fig. S1a-b and Table S1a-b). The lower Rct value of CdFe_2_O_4_ indicates better conductivity and electron mobility. This also might be responsible for its excellent OER activity.

### Hydrogen evolution reaction (HER) activity of CdFe_2_O_4_ catalyst

**Figure 5:**
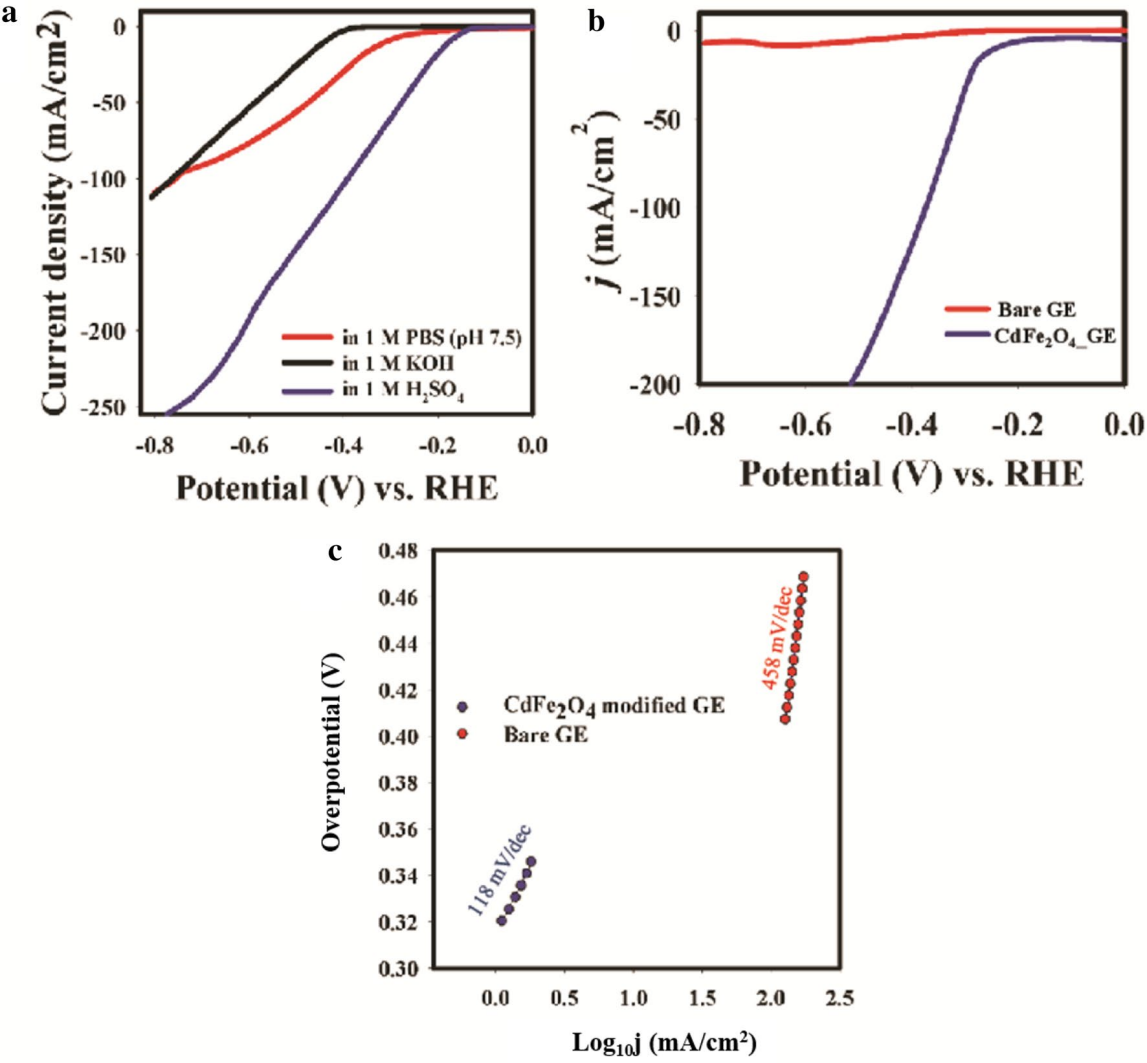
(**a**) effect of supporting electrolyte on HER reaction. (**b**) control study for HER reaction. (**c**) HER Tafel slope.

The HER study was conducted using linear sweep voltammetry and the obtained voltammograms are presented in Fig. 5.

The optimization of hydrogen evolution catalysis by CdFe_2_O_4_ was conducted by varying the pH of the aqueous medium (water). The onset potential for HER reaction occurred at the lowest onset potential in the acidic medium (− 0.2 V vs. RHE) as compared to in 1 M KOH (− 0.41 V) and 1 M PBS (− 0.30 V vs. RHE) solutions (Fig. 5a). CdFe_2_O_4_ exhibited high current density at a very low potential in acidic medium. The conventional 10 mA/cm^2^, corresponding to TOF of 0.02 s^−1^, was achieved at 220 mV overpotential with CdFe_2_O_4_ (Fig. 5b). On a bare Au electrode, the HER reaction was initiated at an overpotential above 800 mV. The HER electro kinetics were assessed with Tafel plots (Fig. 5c). In general, HER in acidic medium follows Volmer-Heyrovsky model involving transfer of two electrons catalyzed by the active catalysts. In this study, CdFe_2_O_4_ reduce the energy barrier in electron transfer reaction. The mechanism for this reaction is as follows:

- Volmer step (Tafel slope of ~ − 120 mV/dec): This involves release of hydroxonium ion and adsorption of intermediate Had* on the active sites of CdFe_2_O_4_
  - H_3_O^+^  + [act site] + e^−^ →[H^*^_adsorbed int_] + H_2_O
- Heyrovsky step (Tafel slope of ~ − 40 mV/dec): In this step, the discharged hydroxonium ion reacts with adsorbed intermediate H*ad to generate hydrogen gas
  - H_3_O^+^  + H*[adsorbed int] + e^−^ →[act site] + [H*_ad_ adsorbed int.] + H_2_ + H_2_O
- Tafel step (Tafel slope of ~ − 30 mV/dec): This reaction is governed by diffusion with faster kinetics.
  - 2H[adsorbed int] → H_2_+[active site]

In the current study, the Tafel slope of CdFe_2_O_4_ was 118 mV/dec corresponding to Volmer step rate determining reaction. Compared with unmodified GE with Tafel slope of 800 mV/dec., CdFe_2_O_4_ greatly facilitated the HER evolution while that of unmodified GE depicts a kinetically impaired reaction. Compare with the literature, CdFe_2_O_4_ compares well with HER performance (Table S3b).

### Catalyst stability test

**Figure 6:**
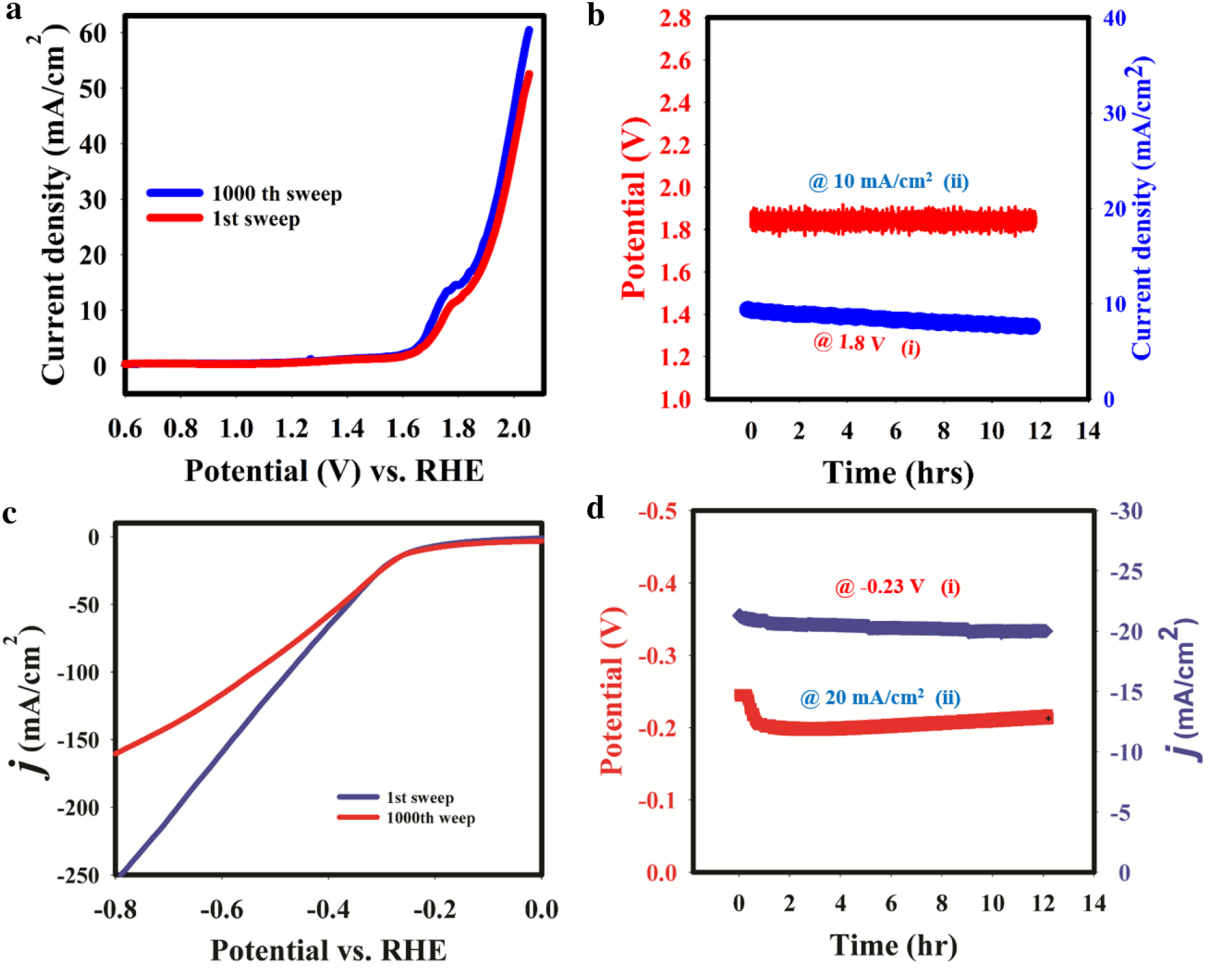
(**a**) OER sweeps (1000th cycles). (**b**) chronoamperometric and chronopotentiometric stability studies for OER. (c) HER sweeps (1000th cycles). (**d**) chronoamperometric and chronopotentiometric studies for HER.

The developed CdFe_2_O_4_ electrocatalyst was subjected to chronoamperometric, chronopotentometric and multiple linear sweep studies. The obtained chronoamperograms, multiple linear sweeps and chronoamperograms for OER reactions are displayed in Fig. 6a,b.

The developed catalyst displayed high stability with current degradation of just 3% (Fig. 6bi); while the potential drop and linear sweep current density were 30% (Fig. 6bii) and 11% (Fig. 6a) respectively. For HER study, CdFe_2_O_4_ exhibited high stability in acidic medium with current density and potential degradation of 10% (Fig. 6di) and 20% (Fig. 6dii) respectively. Also, after 1000th cycle, the linear sweep current density only dropped by 2% at the current density of 10 mA/cm^2^ (Fig. 6c). These data indicate that CdFe_2_O_4_ is highly stable for OER and HER reactions especially at the current density of 10 mA/cm^2^.

## Conclusion

This study demonstrated facile synthesis of bifunctional electrocatalyst based on the mixed oxide CdFe_2_O_4_. The newly developed material was characterized and applied for electrochemical water splitting. The synergistic effect through sharing of active sites and electrochemically surface area of the composite material ensures good electrocatalytic property of the resulting composite, CdFe_2_O_4_. For HER reaction, the onset potential for the reaction was − 0.2 V with overpotential of 220 mV (to reach 10 mA/cm^2^) and Tafel slope of 118 mV/dec in acidic medium. The moderate Tafel slope indicates that the HER reaction proceeded by a Heyvsroky process. In addition, for OER reaction, the onset potential was 1.6 V, with overpotential of 0.47 V and Tafel slope of 104 mV/dec in alkaline medium. The developed catalyst displayed high catalytic stability with very low current density and potential drop in both OER and HER reactions after continuous use for up to 12 h. This study therefore presents a low cost approach for OER and HER catalyst synthesis for potential hydrogen gas generation to support clean fuel production and sustainable development.

## Supplementary Information

Supplementary Information.
