# Supplementary material for: Solid-state synthesis of CdFe2O4 binary catalyst for potential application in renewable hydrogen fuel generation
Source: Sci Rep. 2022 Jan 31;12:1632. doi: 10.1038/s41598-022-04999-1 (PMC8803981; doi:10.1038/s41598-022-04999-1)
Supplement: Supplementary file 1 — Supplementary Information. [file 41598_2022_4999_MOESM1_ESM.doc]

**Electronic Supporting Information**

**Solid-state synthesis of CdFe_2_O_4_ binary catalyst for potential application in**

**renewable hydrogen fuel generation**

Abdullah M. Asiri^1,2^, Waheed A. Adeosun^2^, Sher Bahadar Khan^1,2^, Khalid A Alamry^2^, Hadi M. Marwani^2^, Shaik M. Zakeeruddin^3^, Michael Grätzel^3^

*^1^Center of Excellence for Advanced Materials, King Abdulaziz University, P.O. Box 80203, Jeddah, Saudi Arabia 21589*

*^2^Department of Chemistry, King Abdulaziz University, P.O. Box 80203, Jeddah, Saudi Arabia 21589*

*^3^Laboratory of Photonics and Interfaces, École Polytechnique Fédérale de Lausanne, 1015 Lausanne, Switzerland*

Correspondence: Prof. Abdullah M. Asiri, [aasiri2@kau.edu.sa](mailto:aasiri2@kau.edu.sa)

Prof. Michael Grätzel, michael.graetzel@epfl.ch

S1 **Electrochemical Impedance Spectroscopy (EIS) study**

The circuit diagrams for bare GE and CdFe_2_O_4_ modified gold electrode are given below.

Please explain the meaning of the various symbols used in all figures and tables.


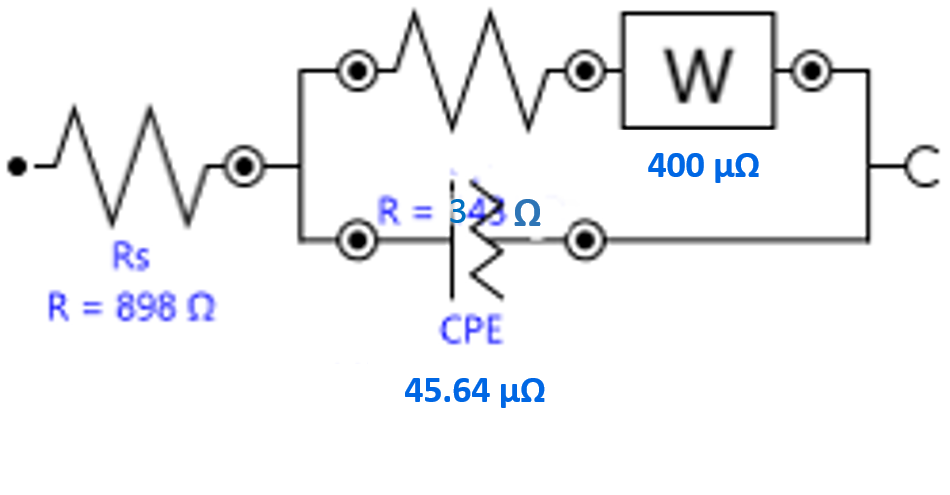


Fig. S1a: Equivalent circuit diagram for the EIS study (bare GE in 1 M KOH).

Table S1a: Values of electrochemical circuit parameters for bare GE

| Element | Parameter | Value |
| --- | --- | --- |
| Rs (Ω) | R | 898.18 |
| Rp (Ω) | R | 343 |
| CPE (µΩ) | Y0 | 45.64 |
|  | N | 0.99838 |
| W(µΩ) | Y0 | 400 |

*CPE-constant phase element; W – Warbug impedance; Rs-solution resistance; Rp-polarization resistance.


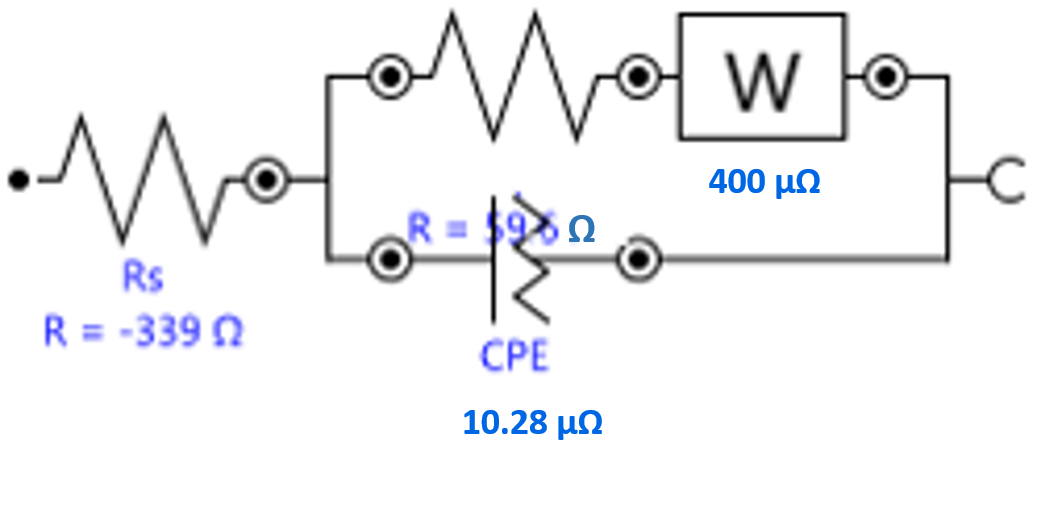


Fig. S1b: Equivalent circuit diagram for the EIS study (CdFe_2_O_4_ modified GE in 1 M KOH).

Table S1b: Values of electrochemical circuit parameters for CdFe_2_O_4_ modified GE

| Element | Parameter | Value |
| --- | --- | --- |
| Rs (Ω) | R | 339.19 |
| Rp (Ω) | R | 59.61 |
| CPE (µΩ) | Y_0_ | 10.28 |
|  | N | 0.99546 |
| W (µΩ) | Y0 | 400 |

S2. Faradaic efficiency of oxygen


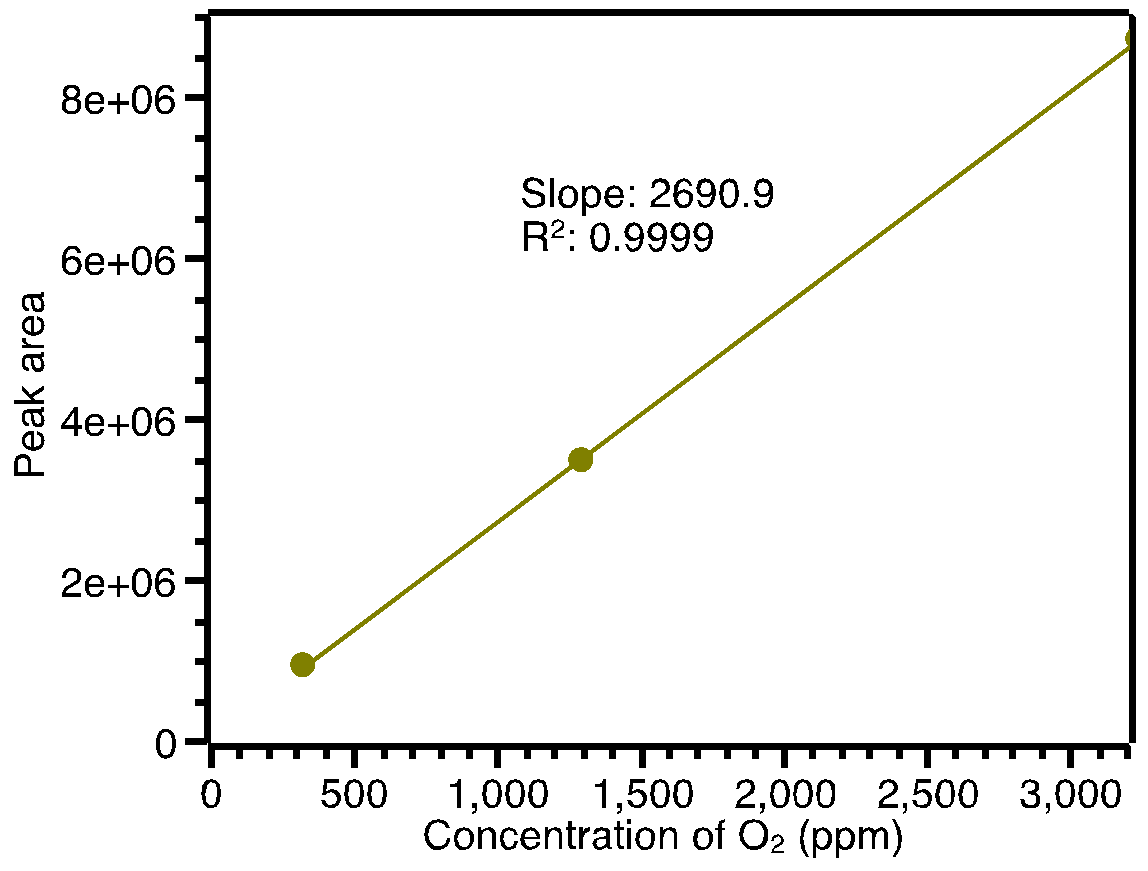


Figure S2. Calibration curve of oxygen for gas chromatography.

Table S2. Faradaic efficiency of oxygen using CdFe_2_O_4_ catalysts at 10 mA cm^-2^ for two hours.

| Sampling time (s) | Concentration (ppm) | Faradaic efficiency |
| --- | --- | --- |
| 984 | 1002.7 | 99.87% |
| 1824 | 998.0 | 99.40% |
| 2664 | 968.9 | 96.50% |
| 3504 | 1016.4 | 101.23% |
| 4344 | 989.5 | 98.55% |
| 5184 | 1029.2 | 102.50% |
| 6024 | 1030.5 | 102.63% |
| 6864 | 986.1 | 98.21% |
| Average |  | 99.86% |

S3: Comparison of performance of CdFe_2_O_4_ modified GE with the previously reported OER and HER catalysts

Table S3a: Previously reported OER catalysts as compared with CdFe_2_O_4_ catalyst

| **OER catalyst** | **Overpotential**  **(V)**  **to 10 mA/cm^2^** | **Tafel slope (mV/dec)** | **Supporting electrolyte** | **Onset potential (V)** | **Ref.** |
| --- | --- | --- | --- | --- | --- |
| 2D/3D Si-MnO_2_ | 0.61 | 60 | 0.1 M KOH | 1.6 | [1] |
| 3D α-MnO_2_ | 0.64 | 155 | 0.1 M KOH | 1.65 | [2] |
| CoFe_2_O_4_ composite | 0.54 | 130 | 0.1 M KOH | 1.64 | [3] |
| Pt/Carbon black based catalyst | 0.47 | - | 0.1 M KOH | 1.63 | [4] |
| NiO-PANI composite based catalyst | 0.75 | 42 | 0.1 M KOH |  | [5] |
| MoFs derived NiFeP | 0.23 | - | 1 M KOH | 1.53 | [6] |
| ZIF-67@CoFe-PBA-F-250 | 0.24 | 48 | 1 m KOH | 1.45 | [7] |
| CdFe_2_O_4_ modified GE | 0.47 V | 103 | 1 M KOH | 1.6 | This work |

Table S3b: Previously reported HER catalysts as compared with CdFe_2_O_4_ catalyst

| HER catalyst | Ƞ to 10 mA/cm^2^ | Tafel slope (mV/dec) | Electrolyte | Onset potential (mV) | Ref |
| --- | --- | --- | --- | --- | --- |
| Rh_2_-Ir alloy | 12 mV  9 mV | 17.3 | 1 m HClO_4_  1 M KOH | -0.015 | [8] |
| Biomass-based carbon | 133 | 78 | 1 M KOH | - 200 | [9] |
| Pt/Zn_3_P_2_/Nickel foam | 74 mV | 55 | 1 M KOH | -700 | [10] |
| MoFs derived NiFeP | 32 mV | 80 | 1 M KOH | -200 | [6] |
| Au-NiSx | 253 mV | 43.7 | 0.5 M H_2_SO_4_ | -220 | [11] |
| CoFeNiMo@NCNT | 209.9 mV | 67.4 | 0.5 M H_2_SO_4_ | -190 | [12] |
| CuS/Au | 179 mV | 133 | 0.5 M H_2_SO_4_ | -141 | [13] |
| CdFe_2_O_4_ modified GE | 220 | 118 | 1 M H_2_SO_4_ | -200 | ***This work*** |

References

[1] K. Selvakumar, S.M.S. Kumar, R. Thangamuthu, P. Rajput, D. Bhattacharyya, S.N. Jha, 2D and 3D Silica-Template-Derived MnO2 Electrocatalysts towards Enhanced Oxygen Evolution and Oxygen Reduction Activity, ChemElectroChem. 5 (2018) 3980–3990. doi:https://doi.org/10.1002/celc.201801143.

[2] X. Zheng, L. Yu, B. Lan, G. Cheng, T. Lin, B. He, W. Ye, M. Sun, F. Ye, Three-dimensional radial Α-MnO2 synthesized from different redox potential for bifunctional oxygen electrocatalytic activities, J. Power Sources. 362 (2017) 332–341. doi:10.1016/j.jpowsour.2017.07.027.

[3] K. Xie, J. Masa, E. Madej, F. Yang, P. Weide, W. Dong, M. Muhler, W. Schuhmann, W. Xia, Co3O4–MnO2–CNT Hybrids Synthesized by HNO3 Vapor Oxidation of Catalytically Grown CNTs as OER Electrocatalysts, ChemCatChem. 7 (2015) 3027–3035. doi:https://doi.org/10.1002/cctc.201500469.

[4] Z.Y. Li, S.T. Shi, Q.S. Zhong, C.J. Zhang, C.W. Xu, Pt-Mn3O4/C as efficient electrocatalyst for oxygen evolution reaction in water electrolysis, Electrochim. Acta. 146 (2014) 119–124. doi:10.1016/j.electacta.2014.09.067.

[5] J. He, M. Wang, W. Wang, R. Miao, W. Zhong, S.-Y. Chen, S. Poges, T. Jafari, W. Song, J. Liu, S.L. Suib, Hierarchical Mesoporous NiO/MnO2@PANI Core–Shell Microspheres, Highly Efficient and Stable Bifunctional Electrocatalysts for Oxygen Evolution and Reduction Reactions, ACS Appl. Mater. Interfaces. 9 (2017) 42676–42687. doi:10.1021/acsami.7b07383.

[6] X. Xu, T. Wang, C. Zhao, Z. Huang, M. Zheng, R. Jia, Y. Liu, MOFs derived NiFeP porous nanoflowers for boosted electrocatalytic water splitting, Microporous Mesoporous Mater. 312 (2021) 110760. doi:10.1016/j.micromeso.2020.110760.

[7] X. Gu, Z. Liu, H. Liu, C. Pei, L. Feng, Fluorination of ZIF-67 framework templated Prussian blue analogue nano-box for efficient electrochemical oxygen evolution reaction, Chem. Eng. J. 403 (2021) 126371. doi:10.1016/j.cej.2020.126371.

[8] Q. Liu, C. Fan, X. Zhou, J. Liu, S. Jiang, S. Wang, X. Wang, Y. Tang, Facile fabrication of hierarchical Rh2Ir alloy nanodendrites with excellent HER performance in a broad pH range, New J. Chem. 44 (2020) 21021–21025. doi:10.1039/d0nj04512h.

[9] O. Abiola Fakayode, B. Adegbemiga Yusuf, C. Zhou, Y. Xu, Q. Ji, J. Xie, H. Ma, Simplistic two-step fabrication of porous carbon-based biomass-derived electrocatalyst for efficient hydrogen evolution reaction, Energy Convers. Manag. 227 (2021) 113628. doi:10.1016/j.enconman.2020.113628.

[10] L.N.T. Mai, T.C. Lam, Q.B. Bui, H.T. Nhac-Vu, Efficient hydrogen evolution reaction in alkaline via novel hybrid of Pt deposited zinc phosphide nanosheets, Mater. Res. Bull. 133 (2021) 111024. doi:10.1016/j.materresbull.2020.111024.

[11] Y. Lv, S. Duan, Y. Zhu, H. Guo, R. Wang, Interface control and catalytic performances of Au-NiSx heterostructures, Chem. Eng. J. 382 (2020) 122794. doi:10.1016/j.cej.2019.122794.

[12] T. Wang, M. Xu, F. Li, Y. Li, W. Chen, Multimetal-based nitrogen doped carbon nanotubes bifunctional electrocatalysts for triiodide reduction and water-splitting synthesized from polyoxometalate- intercalated layered double hydroxide pyrolysis strategy, Appl. Catal. B Environ. 280 (2021) 119421. doi:10.1016/j.apcatb.2020.119421.

[13] M. Basu, R. Nazir, P. Fageria, S. Pande, Construction of CuS/Au Heterostructure through a Simple Photoreduction Route for Enhanced Electrochemical Hydrogen Evolution and Photocatalysis, Sci. Rep. 6 (2016) 1–11. doi:10.1038/srep34738.
